# Area of origin analysis for expirated patterns using HemoVision

**DOI:** 10.1111/1556-4029.70375

**Published:** 2026-06-04

**Authors:** Lathursha Kalaranjan, Eugene Liscio

**Affiliations:** ^1^ University of Toronto Mississauga Mississauga Ontario Canada; ^2^ ai2‐3D Forensics Ontario Canada

**Keywords:** area of origin (AO), bloodstain pattern analysis (BPA), coughing mechanism, crime scene reconstruction, expirated spatter pattern, forensic identification, HemoVision software, misting pattern, virtual stringing method

## Abstract

Although bloodstain pattern analysis (BPA) can be performed on various types of patterns, there is a lack of research looking into whether the area of origin (AO) can be determined for expirated patterns. Expirated patterns are formed when blood is pushed out of an opening in the body by the force of air, in some cases forming a pattern of radiating bloodstains which can be analyzed similarly to impact patterns. This research aims to compare the true versus observed AO of expirated patterns using a mechanical rig. In addition, a series of blind expirated patterns was created by human participants. The resultant patterns were analyzed in HemoVision to deduce the AO and determine what kinds of errors manifest. Using the rig, five expirated patterns were produced from 20 cm and 40 cm from a target, at 90° and 45° towards the wall (*n* = 20). The errors in each direction (X, Y, Z) were assessed. One‐sample *t*‐tests of the 3D coordinates found statistically significant differences between the sampled and hypothesized mean (*μ* = 0) at 90° and 20 cm (*x* = 11.89, *p* = 7.220E‐04), 90° and 40 cm (*x* = 22.54, *p* = 5.764E‐06), 45° and 20 cm (*x* = 13.41, *p* = 9.888E‐04), and 45° and 40 cm (*x* = 28.16, *p* = 4.040E‐05). This indicates that the AO may not be accurately calculated for expirated patterns, with the largest difference being the distance from the wall. This suggests a unique limitation compared to impact patterns, which tend to have the greatest errors in height. Blind tests were also analyzed to determine the quality of AO analysis using samples from human participants (*n* = 11).

**Highlights**

- Expirated patterns from high‐velocity coughing exhibit features affecting AO estimation.
- Fluid type, volume, velocity, orifice, and human factors limit AO estimation.
- *X* and *Z* estimates support two‐dimensional AOC for investigative purposes.
- *Y*‐axis errors present limitations in accurately estimating the three‐dimensional AO.

## 1 INTRODUCTION

Bloodstain pattern analysis (BPA) is a forensic discipline that utilizes the principles of physics and visual examination techniques to analyze patterns formed by blood at a crime scene. The core of BPA involves the examination of the size, shape, location, and orientation of blood spatter to derive the path of travel [1]. The resultant findings can provide information regarding the physical events leading to the dispersion of the blood, allowing investigators to perform crime scene reconstruction [1].

One of the most common analyses that may be considered through BPA is the area of origin (AO), which is the three‐dimensional space from which blood originated before it was dispersed through air and onto the surface of interest [2, 3]. The AO can help infer the original position of a bleeding individual, a blood‐coated object, or the placement of a volume of blood. In the case of expirated patterns, the AO would primarily refer to the position of the individual, the source of their injury, and, more specifically, the position of the orifice from which blood is dispersed. Analysis of the AO relays information regarding the distance, direction, and angle at which the blood traveled before impact with the surface. This information is significant for the purpose of reconstructing the events of the crime, validating testimonies, and understanding the nature of the crime [3]. Thus, BPA can be used to determine information that can establish the chain of events prior to blood dispersion, which is significantly useful for forensic analysis and crime scene investigation.

AO analysis is performed by analyzing individual bloodstains to determine the angle and direction of travel, which can also provide the area of convergence (AOC). The AOC is the two‐dimensional space in a plane that indicates the directionalities of the converging spatter trajectories [2]. The three‐dimensional AO requires the distance between the surface and the origin position, which is assumed to be perpendicular to the AOC. This distance is determined by the impact angle and how all the blood drops converge in 3D space. The angle of impact is calculated based on the arcsine of the length and width of the elliptical bloodstain [4, 5].

Longer and more elliptical bloodstains are preferred over circular bloodstains. Blood has a high surface tension and viscosity, which causes the platelets of the blood to gather, thereby forming a spherical blood drop [6]. Depending on the direction and force of travel, this spherical blood drop can create two distinct shapes once it comes in contact with a surface: circular and elliptical stains. Circular bloodstains are characterized by a round shape and lack of tail, which indicates that the blood drop may have traveled perpendicularly to the surface. Elliptical bloodstains are characterized by a tail, which is indicative of a non‐perpendicular travel. Elliptical spatter provides significant information about the directionality of travel, considering that the leading edge of the stain shows the initial point of contact, and the tail of the stain, should it exist, shows the final location of the stain, which is formed upon impact with the surface [7]. As a result, the tail is used to determine the direction that the blood traveled prior to contact with the surface, while the length and width of the stain provide further information about the angle of impact.

One of the most well‐known and oldest methods of AO analysis is the stringing method—an accepted method in which strings are attached to the ends of various bloodstains and traced back to the AOC [8, 9, 10]. The tangent method measures the dimensions of the stain and uses trigonometry to trace back to a three‐dimensional AO coordinate [9]. A major limitation of physical methods is their invasive techniques, such as the attachment of strings to a number of bloodstains found at the crime scene, which may further be seen as a physically cumbersome task [4, 10]. As a result, automated methods of AO analysis through software are being developed to perform digital crime scene reconstruction, allowing for virtual methods of BPA.

One such software is HemoVision, produced by Forentrics, which employs automated methods through machine learning and computer vision to analyze blood spatter [9, 11]. HemoVision uses an optimization model through mathematical functions built upon the tangent method to return an AO estimation based on the likelihood of convergence [9, 11]. HemoVision requires the bloodstain pattern to be marked with image markers that account for scale, orientation, and perspective distortions. Photographs are taken from various positions at a wide angle and close range. The photographs are loaded into the software, which then performs an automatic 3D reconstruction of the scene based on an overall reference marker (orienting the walls) and smaller detail markers (orienting the individual bloodstains). As opposed to the stringing method, HemoVision requires the general placement of small image markers near droplets of interest, along with a general position marker, and thus, may be viewed as less physically cumbersome. Once reconstructed, the stains of interest can be virtually marked, and HemoVision can estimate the AO based on the highlighted stains. This computational software builds upon the stringing method by introducing virtual stringing, allowing for remote and non‐invasive AO analysis. The virtually rendered AO analysis produced by HemoVision allows for a visualization of individual trajectories and the general AO, providing a visualization and numerical value for margins of error for each vector. Though it should be noted that, unlike traditional stringing methods, computational software introduces financial burdens associated with download costs. Further, software is not void of human bias, considering the trained algorithms are designed by humans and thus may be subject to human bias [10].

A difficulty associated with expirated pattern analysis is the diverse array of characteristics that describe this subtype of blood spatter. Depending on the orifice from which the blood exits, there is a possibility for other liquids to be mixed with the blood—such as saliva, phlegm, or mucus if the blood is expelled from the mouth or nose. Further, the force exerted on the blood drives it through the air, against the pressure of air resistance, thus resulting in unique patterns. For example, a heavy coughing motion can result in airborne or mist‐like patterns, whereas a spitting motion can result in string‐like patterns [12]. For this study, the orifice considered is the human mouth, and the force of air is defined as a coughing motion.

The coughing motion is created by airflow that travels up the trachea and out of the mouth. The human trachea is a semi‐cylindrical airway that is roughly 10–20 cm long with an internal diameter of 1.6–2.5 cm [13, 14, 15]. The diameter of the trachea is positively related to the pressure exerted by the airflow. Other factors that can influence the expansibility of the trachea include age and the presence of pulmonary disease [14, 16]. Thus, this study aims to focus on expirated patterns created by adult‐aged individuals who are not affected by pulmonary disease.

A human cough can aerodynamically be characterized based on the volume of air and velocity of travel, both of which can influence the resulting expirated pattern. There are two primary types of coughs: reflexive, which is triggered by disruptions in the airway, and voluntary, which is triggered on command. Voluntary coughs have been found to display greater lung volume initiation and excursion and thus can result in a greater volume of air being expirated [17, 18]. The volume of air, however, is influenced by the inspiratory phase, which depends on the volume of air being breathed in as well as the total lung capacity. The lung capacity is influenced primarily by the individual's height and can also differ based on population group, size, and age [19, 20]. The peak flow, or velocity at which the air travels, can vary based on age, gender, and height. One such study found that younger individuals (aged 20–49) can average peak flow rates of 803.8 L/min in comparison to older individuals who averaged 604.5 L/min [21]. It was also found that male participants displayed an average peak flow of 828.2 L/min in comparison to female participants who averaged 545.1 L/min [21].

The purpose of this research is to examine expirated patterns and compare the true AO to the estimated AO using HemoVision. This research is significant as it will establish whether investigators can appropriately apply AO analysis to expirated patterns in practical case work.

## 2 METHODS

Preliminary tests were performed to define the parameters of an “adult‐aged individual's cough”, as specified for the purpose of this study. The aim of the preliminary tests was to define two specific variables: the volume of air released from a cough and the velocity at which this air travels. Based on previous literature, the expiratory phase can be characterized by a supramaximal expiratory flow with a flow rate of 12 L/s, followed by a lower expiratory flow rate of 3–4 L/s [22]. The volume of air and peak flow can be influenced by body size, height, age, sex, and total lung capacity [17, 18, 19, 20]. Considering that these parameters highly vary across individuals, the purpose of the preliminary tests was to obtain values that would define a minimum range to be met and mimicked by a mechanical test rig. As such, the equipment used to perform the preliminary tests was used to build a mechanical rig to maintain consistency. Further, participants were selected to cover a range of factors that may influence the volume and velocity—such as sex, age, lung size, and overall stature.

**FIGURE 1:**
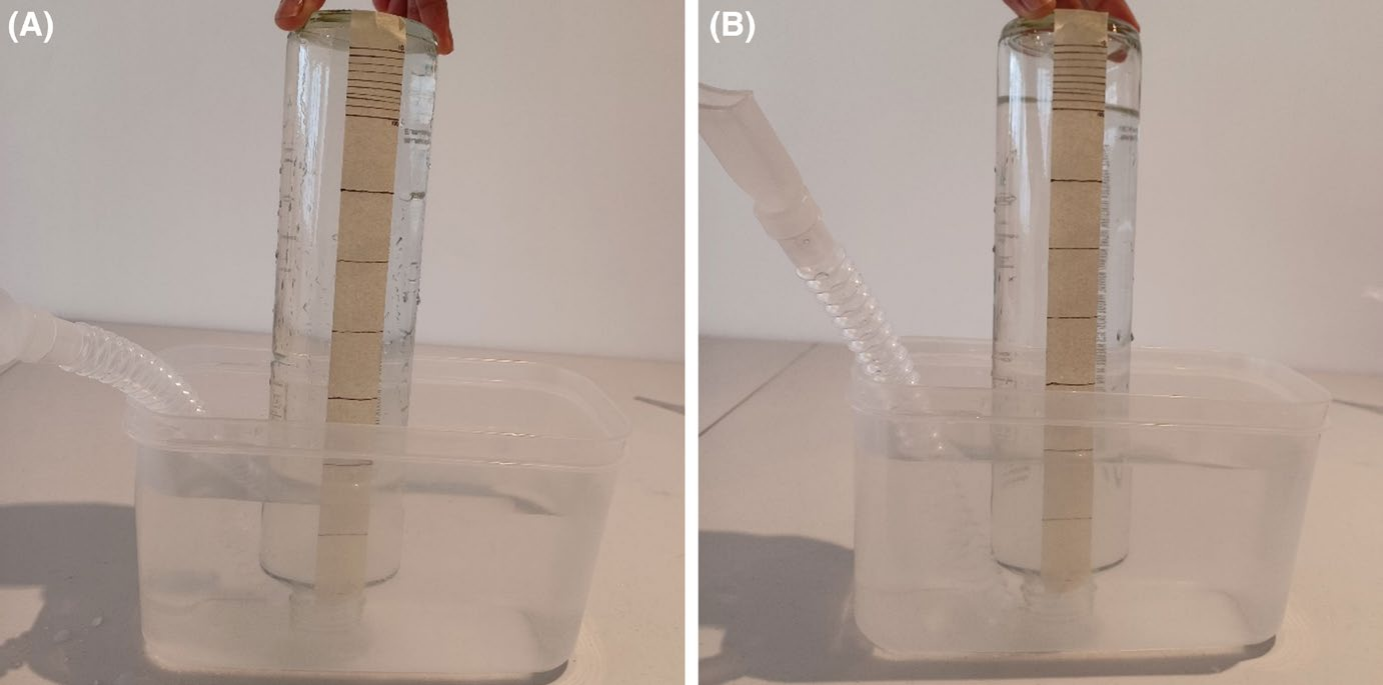
Custom spirometer built to measure the volume of air in a cough. (A) Spirometer before air is expelled through the tube. (B) Spirometer after air has been expelled, thus displacing the water and capturing air (measured in 10 mL intervals).

In terms of volume of air, a custom spirometer was used to measure the amount of water that was displaced based on the amount of air that was expelled (Figure 1). Across ten trials, four adult participants (*n* = 4) coughed into a tube connected to a glass filled with water, thus displacing a volume of water contained within the glass. During expiration, participants stood upright and held the mouthpiece horizontally perpendicular to the glass, mimicking an individual placed at a 90° angle towards a wall. This custom spirometer was used to bypass financial and volume‐related limitations that several commercial spirometers introduced, thus allowing for the measurement of sudden bursts of high‐velocity air. Further, this spirometer could measure volumes of air produced by participants, which were then compared to a mechanical rig upon adjusting the customized mouthpiece. Volumes measured during each trial were noted and photographed before and after each expiration. The resulting volume of air captured within the glass was measured to be the volume of air contained within a mid‐pressure cough from an adult (*V*_avg_ = 77.925 mL).

In terms of the velocity, a 1000 L/H rotameter was used to measure the velocity at which the air traveled in a cough. Across ten trials, two adult participants blew into a 1000 L/H rotameter that was held upwards (*n* = 2). During expiration, participants stood upright and held the rotameter above their head, angling the mouth upwards towards the rotameter. The resulting velocity of air was measured to be the minimum velocity of air captured within a low to mid‐pressure cough from an adult (*v*_avg_ = 557.5 L/H).

**FIGURE 2:**
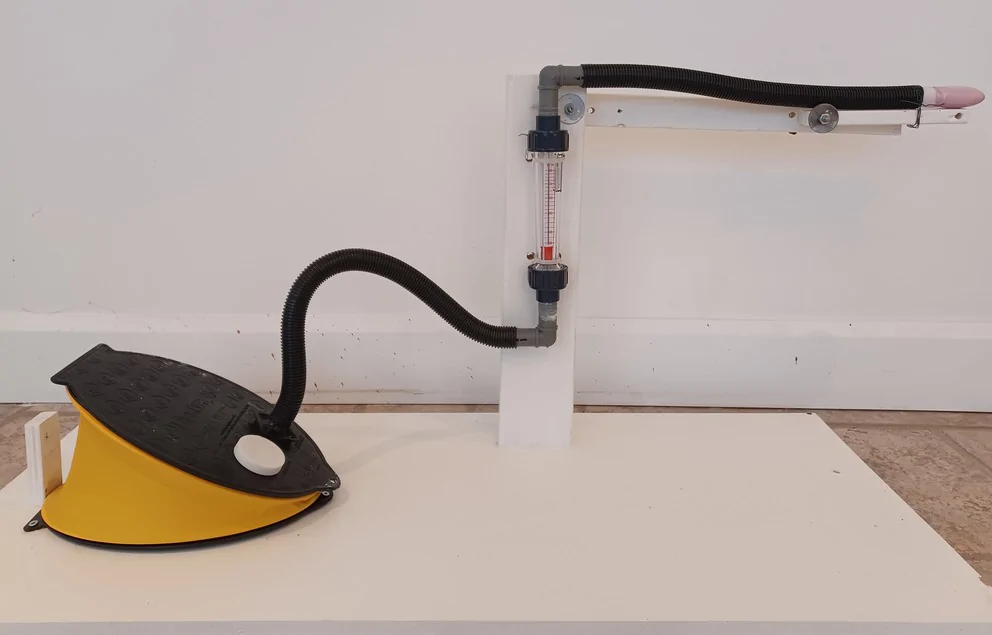
Custom rig built to mimic the coughing motion of an adult‐aged individual.

A mechanical rig was built to produce expirated patterns from a known distance and angle in a repeatable manner (Figure 2). When selecting an air source, the factors that had to be considered were the volume and pressure. For this study, the air source would need to mimic a cough from the mouth of an adult, thereby expelling a high volume of air at a low pressure. Thus, a foot pump was used as the air source as it satisfies the high‐volume, low‐pressure requirement. The foot pump was mounted onto a board that would allow the device to be moved to different distances and angles. A measured slab of wood was mounted directly beside the pump to denote the height to which the pump is to be pushed to meet the average volume determined through the preliminary trials (*V*_avg_ = 77.9 mL). The tube of the air source satisfied the diameter requirement to mimic the trachea of an adult, as it was 2.5 cm in diameter. This tube was secured to a rotameter, which was used as the device that would measure the velocity at which the air travels, to ensure that volumes do not exceed the average determined during the preliminary trials (*v*_avg_ = 557.5 L/H). The rotameter was placed facing upwards and connected to another tube. The end of this tube was secured to a 3D printed mouthpiece that was carefully designed to mimic expirated patterns produced from the mouth of an individual. The tube and mouthpiece were securely fastened to an adjustable arm, which remained perpendicular to the wall at a 90° angle throughout the trials.

Various mouthpiece designs were created and tested until the final design was chosen based on the patterns that were produced when blood was expelled by the air source of the rig. The initial mouthpiece prototypes were created with the intention of mimicking the shape of the mouth, which resulted in spherical pieces that were manually attached to the tube of the rig. The initial prototypes were designed with the intention of mimicking the narrow passage of the mouth when in a coughing position [13, 15]. The narrow passage of the orifice plays a role in coughing dynamics, as high‐velocity air expelled from the lungs encounters resistance when passing through this small passage [13]. This resistance was expected to influence the velocity and trajectory of the expelled blood, thus resulting in unique expirated spatter. Although the initial prototypes held the blood well, the spherical shape produced minimal spatter patterns due to the wide diameter from top to bottom. This diameter resulted in the air being pushed through the spherical mouthpiece without encountering the blood that was held at the bottom of the mouthpiece.

**FIGURE 3:**
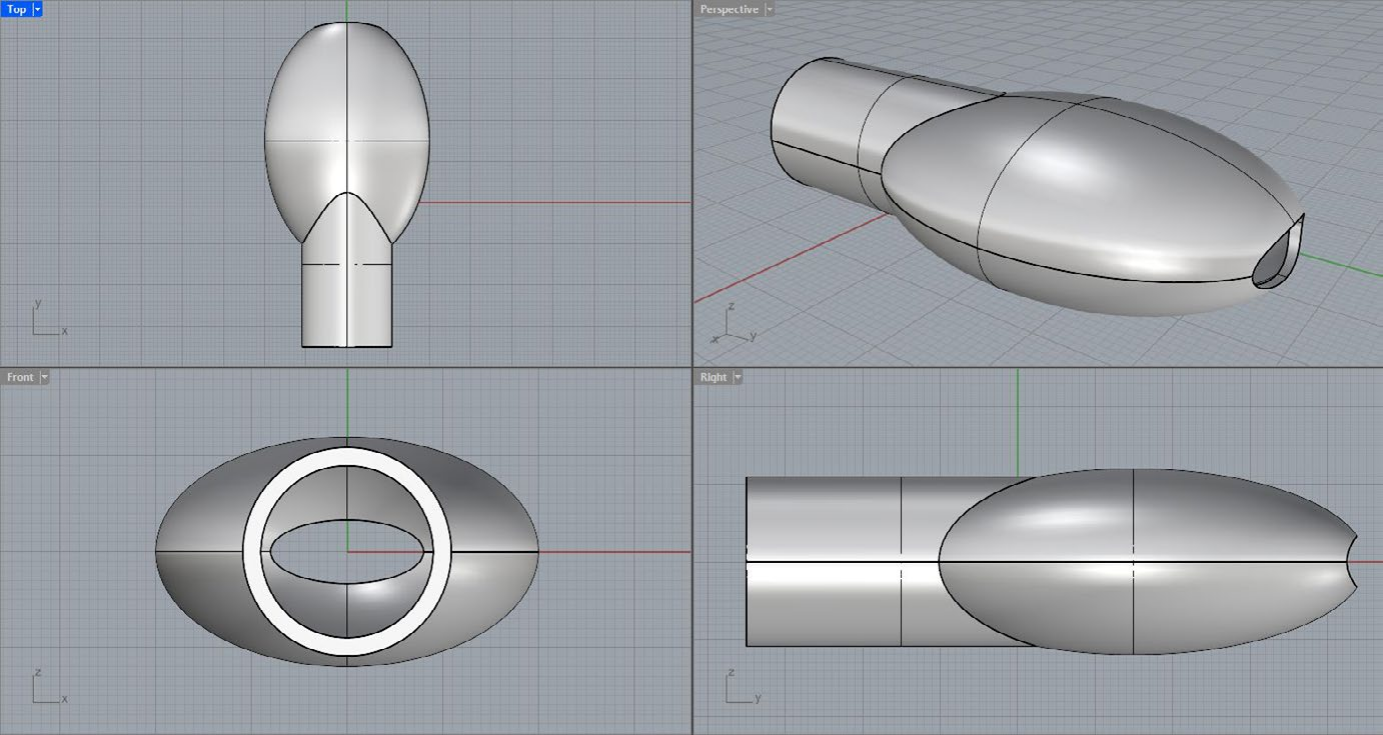
Multi‐view rendering of the design for the 3D‐printed mouthpiece of the rig. From left to right: Top‐down view, angled side view, front view of the mouthpiece opening, and side view.

**FIGURE 4:**
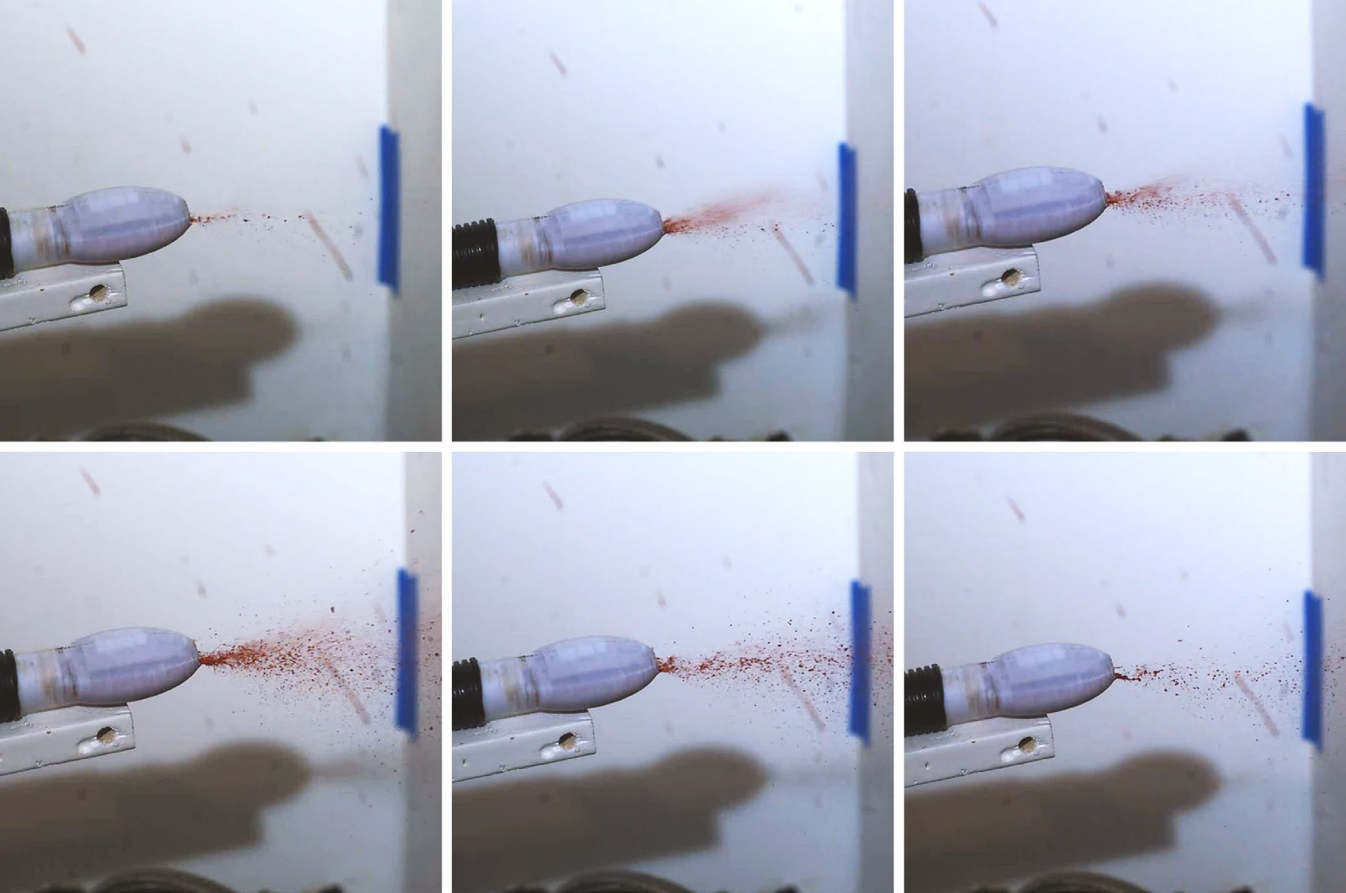
Series of still shots demonstrating the droplets produced by the synthetic mouthpiece, mimicking the shape, size, and radial fashion of expirated patterns (from left to right).

As a result, the prototypes were incrementally modified by flattening the spherical shape from top to bottom. The aim of this design choice was to mimic the tongue, which acts as a bridge between the entrance of the pharynx and the opening of the mouth. This flattening of the mouthpiece was tested repeatedly until the mouthpiece consistently produced spatter patterns mimicking expirated spatter patterns produced by humans, as defined by studies that characterize the dynamics of expirated bloodstain patterns (Figures 3 and 4) [12, 23]. Key characteristics of spatter against a horizontal plane include small mist‐like droplets (<1 mm) followed by large droplets (>1 mm) that form spatter in a radial fashion [12]. Further, the majority of the droplets were to be near‐circular due to the velocities of the spitting or coughing mechanism perpendicular to the plane [23]. In the presence of saliva or mucus, droplets form string‐like patterns; however, the absence of such liquids in the mechanical rig allows for the mimicking of cases in which individuals bleed into airways or cough up blood [12]. As such, the flattened synthetic mouthpiece design was approved for the mechanical rig. The finalized design was developed with a connecting piece that could be attached to the tube to minimize the leakage of air.

The experimental trials were performed with the intent of performing AO analysis on the expirated spatter patterns, to assess whether the calculated and known AO varied based on the angle and distance at which the mouthpiece was positioned from the wall. For this purpose, the rig was to be set up at 20 cm and 40 cm from the wall, and at 90° and 45° from the wall. This resulted in 4 combinations of each distance with each angle, as defined in Table 1. Five trials of expirated patterns were produced at each set (*n* = 20).

**TABLE 1:** Defined sets based on the angle and distance from the rig to the wall.

Set	Angle (°)	Distance from wall (cm)
1	90	20
2	90	40
3	45	20
4	45	40

During each trial, the center of the mouthpiece was measured from a marked point on a paper covering the wall in the X, Z, and Y directions. The three‐coordinate system is well‐defined in AO analysis, within the field of BPA research. The X‐coordinate indicates the horizontal position, in this case, along the wall. The Y‐coordinate indicates the outward position of the origin, in this case, outward from the wall. The Z coordinate indicates the vertical position, in this case, the height of the orifice relative to the ground.

Rig‐based trials were done using sheep blood that was ethically sourced from the Canadian Food Inspection Agency, consistently refrigerated to maintain integrity. For each trial, 5 mL of sheep blood was placed into the mouthpiece using a transfer pipette. The mouthpiece was swirled such that the entirety of the inner mouthpiece was coated with the blood. Once positioned at the proper distance and angle, the foot pump was forced down, and the blood was expirated onto the paper covering the wall. The bottom left corner of the paper was marked, and the center of the mouthpiece was measured from its position to the wall. This position was used as the true location of the AO.

After the patterns dried, the paper was removed from the wall and marked with an overall marker. The overall marker was leveled with the marking on the bottom‐left‐hand corner. The point of measurement on the overall marker was measured from the bottom‐left marking in the X (along the wall) and Z (up and down) directions. The measurement in the Y direction (distance from the wall) was listed as 0 cm because the overall marker was placed flat on the stained paper. Notable stains were pinpointed and marked with small detail markers. The patterns were photographed using a Nikon D7100 DSLR. The photographs were taken at a wide angle, perpendicular to the wall, with minimal angular distortions. This image was to capture the placement of the overall marker, along with the positions of the detail markers relative to this overall marker. Photographs were then taken at a closer range, capturing bloodstains surrounding each detail marker.

**FIGURE 5:**
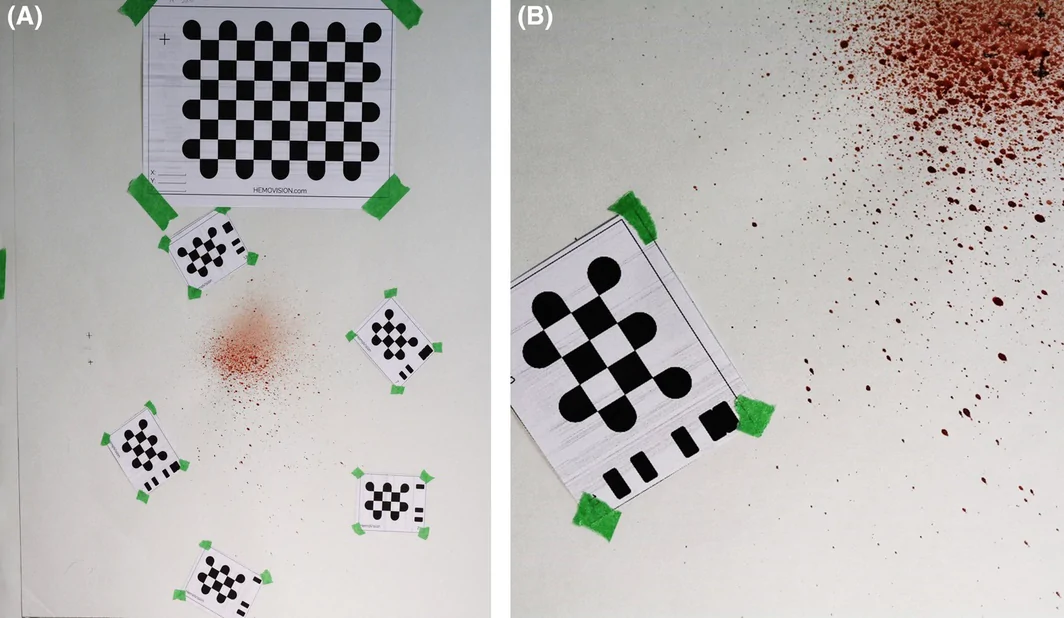
Expirated patterns from the custom rig. (A) General view of expirated patterns with overall and target markers. The point of reference is marked in the bottom left corner. (B) Close‐up view of notable stains near the detail marker.

The wide‐angle images were first loaded into HemoVision, which rendered each target marker relative to the overall marker (Figure 5). The position of the overall marker relative to the marking on the bottom‐left corner of the paper was also input. This was done so that the calculated AO coordinates represent the position of the nozzle relative to the overall marker, as was measured for the true or known coordinates. After the positions of each marker had been rendered in HemoVision, the detail images of the stains surrounding each detail marker were uploaded. The stains were marked on these detail images. The individual stains were marked using the “manual ellipse analysis” tool. This tool involves the manual marking of the length (from the initial point of contact to the final point, excluding the tail) and width of the elliptical stain.

**FIGURE 6:**
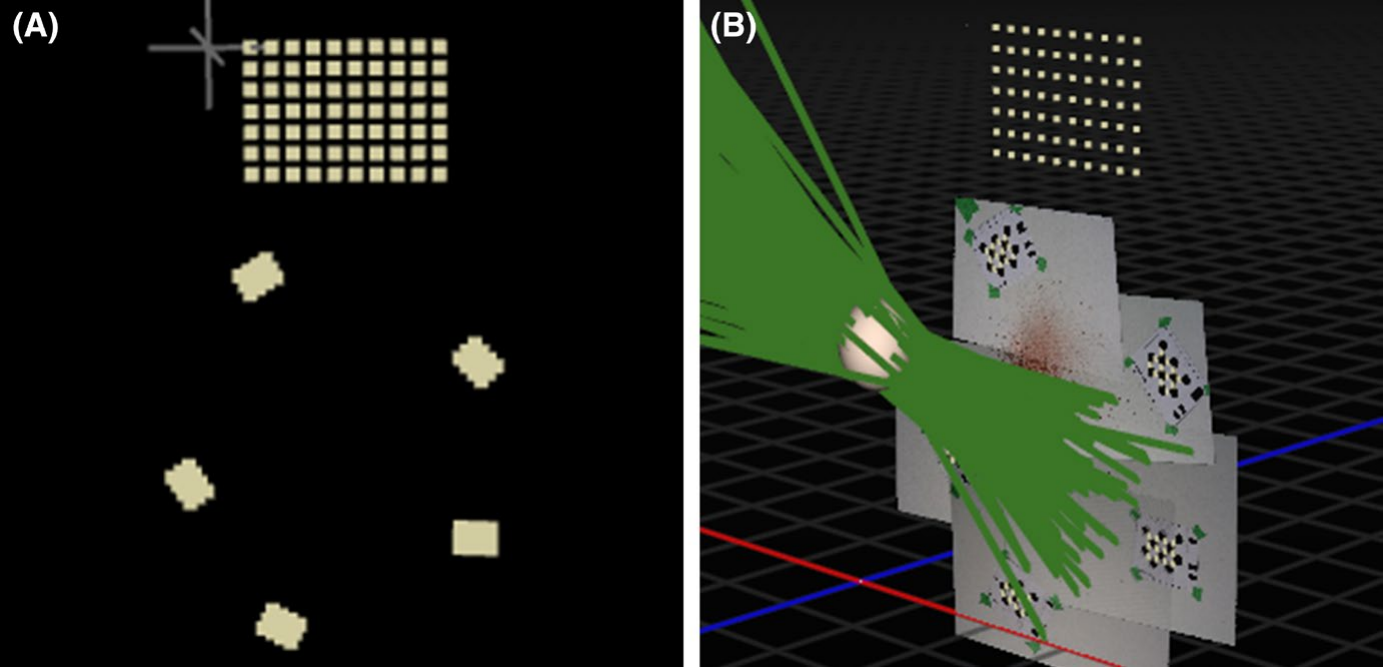
Rendering of the pattern in HemoVision. (A) Rendered based on a wide‐angle photo. (B) Rendering of the AO estimation depicted in white. Projections of the marked stains are depicted in green.

HemoVision uses an automated method to calculate the AO based on the marked elliptical stains. After the stains had been virtually marked, the “area of origin” tool was selected to obtain the position of the virtually calculated AO. This position is returned as the “ellipsoid center,” which represents the center of the AO with the highest possible likelihood of convergence (Figure 6). The AO is reported in the X, Z, and Y directions, all of which were collected and compared to the true values. It should be noted that HemoVision uses an alternative coordinate system, in which the Y‐coordinate indicates the vertical position and the Z coordinate indicates the outward position. For this paper, all coordinates have been corrected to adhere to standard conventions commonly used for AO analysis within BPA research. When comparing the errors, positive values in *X* represented distances closer to the left‐hand side, positive values in Z represented distances closer to the ground, and positive values in the Y represented distances closer to the wall.

**FIGURE 7:**
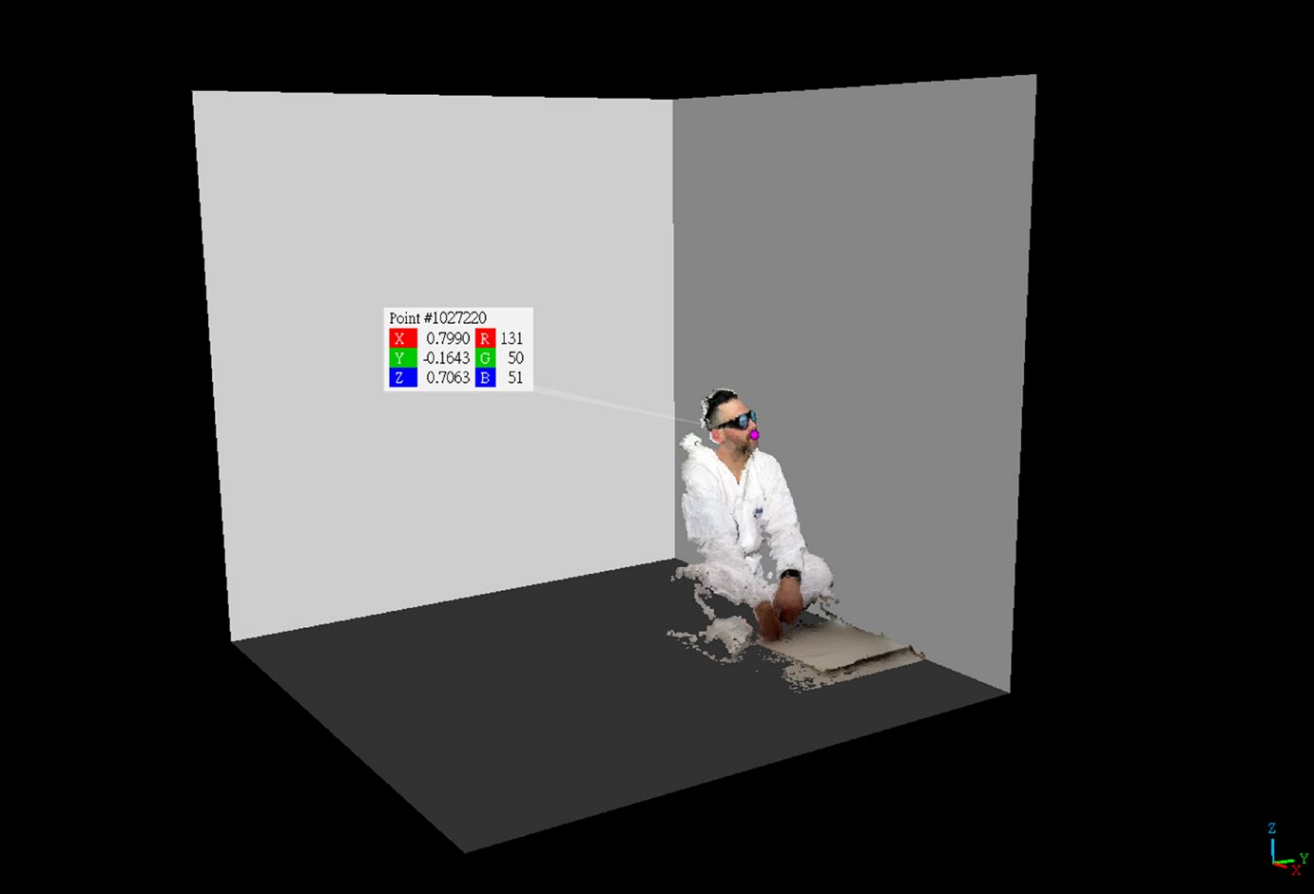
3D scans taken of the participants during the expiration of blood for blind test trials. This image is taken from an angle to the wall. Positions were marked through Cloud Compare and used as the known position for comparison.

To assess the quality of the AO analysis, blind tests were performed and analyzed. Human participants were recruited to place various volumes of their own blood in their mouths and blow at the paper‐covered wall from different distances and angles, unknown to the assessor (*n* = 11). Independent of the assessor, participants were scanned in their positions immediately prior to coughing using the Recon‐3D, a phone‐based LiDAR scanner produced by ai2‐3D, for downstream assessment of the true position. The paper was marked in the bottom‐left corner, which was used as a reference position to be measured from. The resultant patterns were removed from the wall and, similar to the rig tests, marked with an overall marker which was measured from the bottom‐left reference position. The patterns were also marked with detail markers to pinpoint notable stains. The patterns along with their markers were photographed using a D7100 DSLR under optimal lighting at wide and close range. The photographs were loaded into HemoVision and analyzed through the software. As shown in Figure 7, the known positions of the blind tests were recorded using 3D scans, which captured the positions of the participants' mouths in the X, Z, and Y directions as well as the angles at which the participants faced relative to the wall. The 3D scans of each trial were loaded into Cloud Compare, an open‐source 3D point cloud processing software, where the true position was marked as the mouth of the participant. The known coordinates were compared to the coordinates calculated through HemoVision. The errors between the known and calculated positions were assessed to determine the quality of the AO analysis.

## 3 RESULTS

For determining whether the calculated AO was accurate, this was compared to the true coordinates. To make this comparison, the difference of each coordinate in the X, Y, and Z directions was calculated by subtracting the true value from the calculated value. Note that in HemoVision, either the *Z* or *Y* axes may be chosen as the vertical direction based on the analyst's preferred convention. However, in this study, the X direction is the direction along the wall, the Z direction is up and down, and the Y direction is the distance to and from the wall surface. Further, the 3D total error was obtained from the errors in each axis. This was done by calculating the square root of the sum of the coordinates for the errors in each axis, such that

(1) $$T=\sqrt{{\left(x_{t}-x_{c}\right)}^{2}+{\left(y_{t}-y_{c}\right)}^{2}+{\left(z_{t}-z_{c}\right)}^{2}}$$

where *T* in Equation 1 is equal to the three‐dimensional vector of the errors between the true and calculated AO position. The *t* subscripts represent the true value measured during data collection. The *c* subscript represents the calculated value obtained through HemoVision.

### 3.1 Rig tests

**FIGURE 8:**
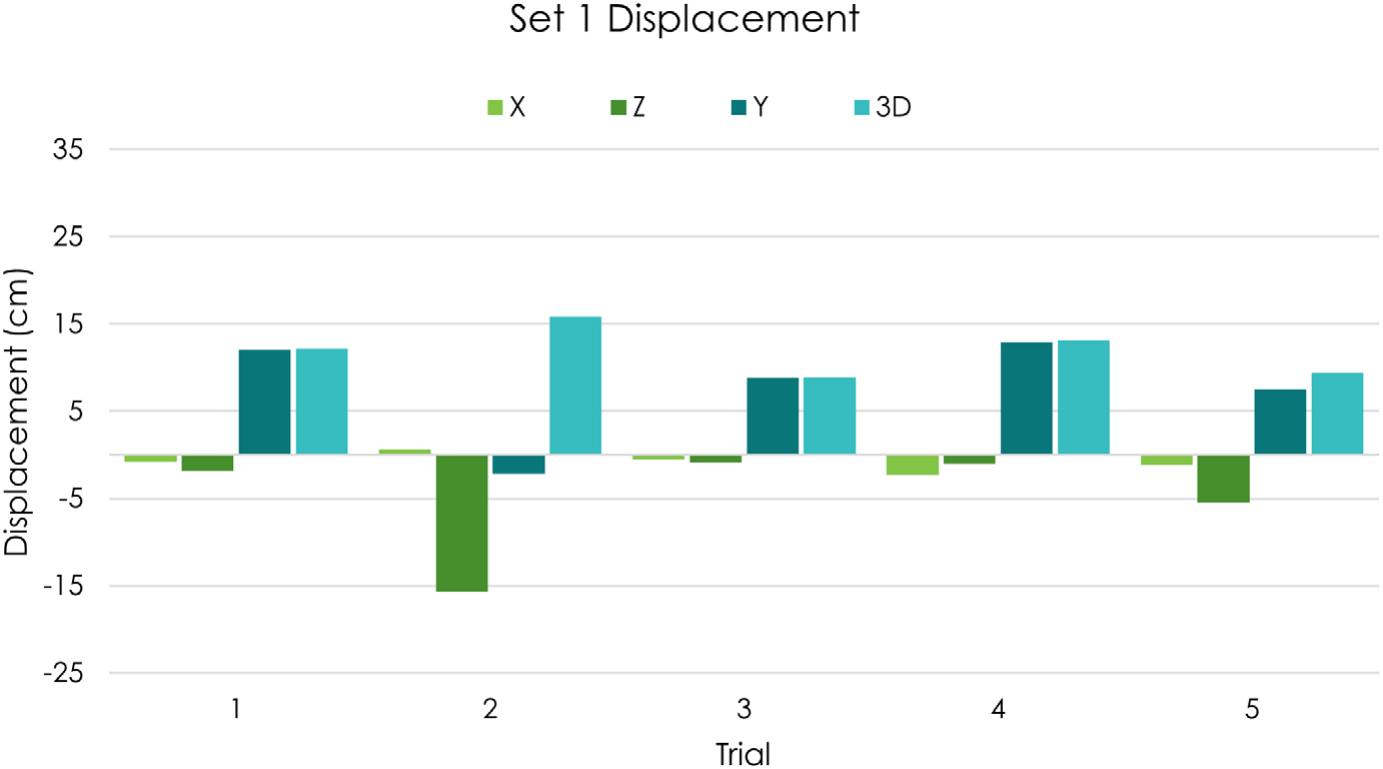
Errors between the known and calculated values for each X, Z, Y, and 3D coordinates. Set 1 is defined as 20 cm from the wall at 90° (perpendicular to the wall).

**FIGURE 9:**
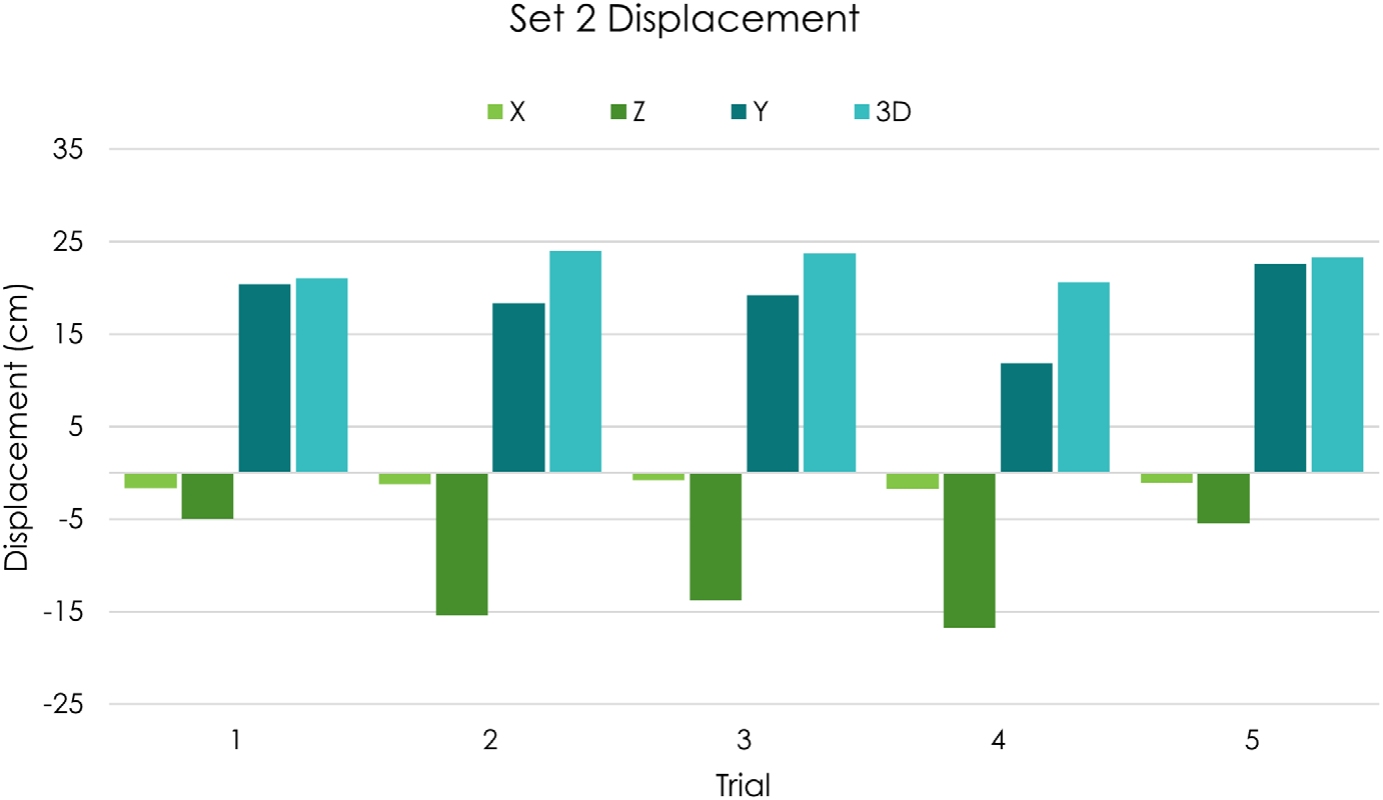
Errors between the known and calculated values for each X, Z, Y, and 3D coordinates. Set 2 is defined as 40 cm from the wall at 90° (perpendicular to the wall).

**FIGURE 10:**
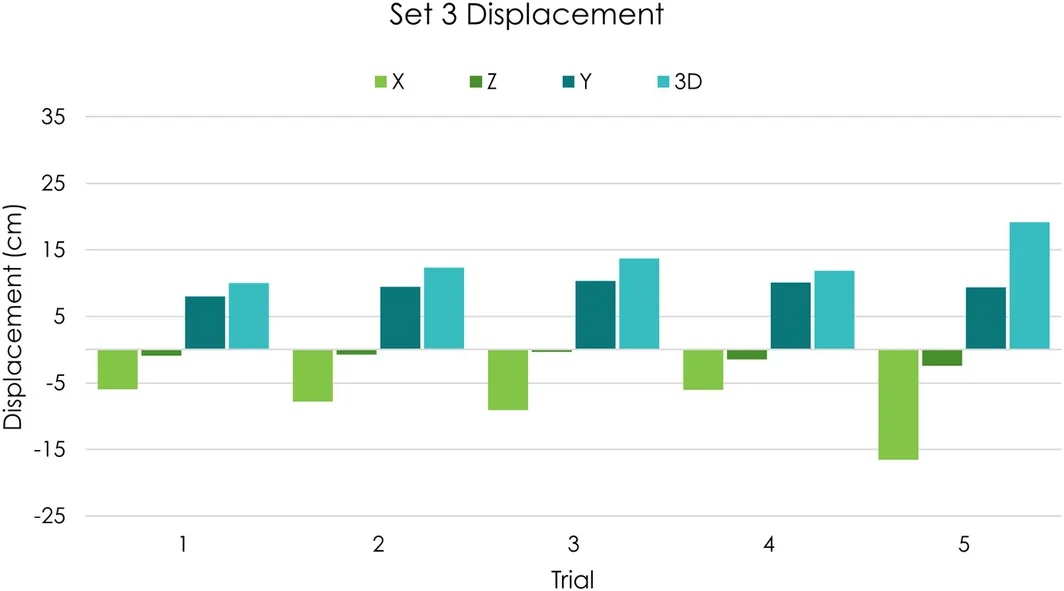
Errors between the known and calculated values for each X, Z, Y, and 3D coordinates. Set 3 is defined as 20 cm from the wall at 45° (angled to the wall).

**FIGURE 11:**
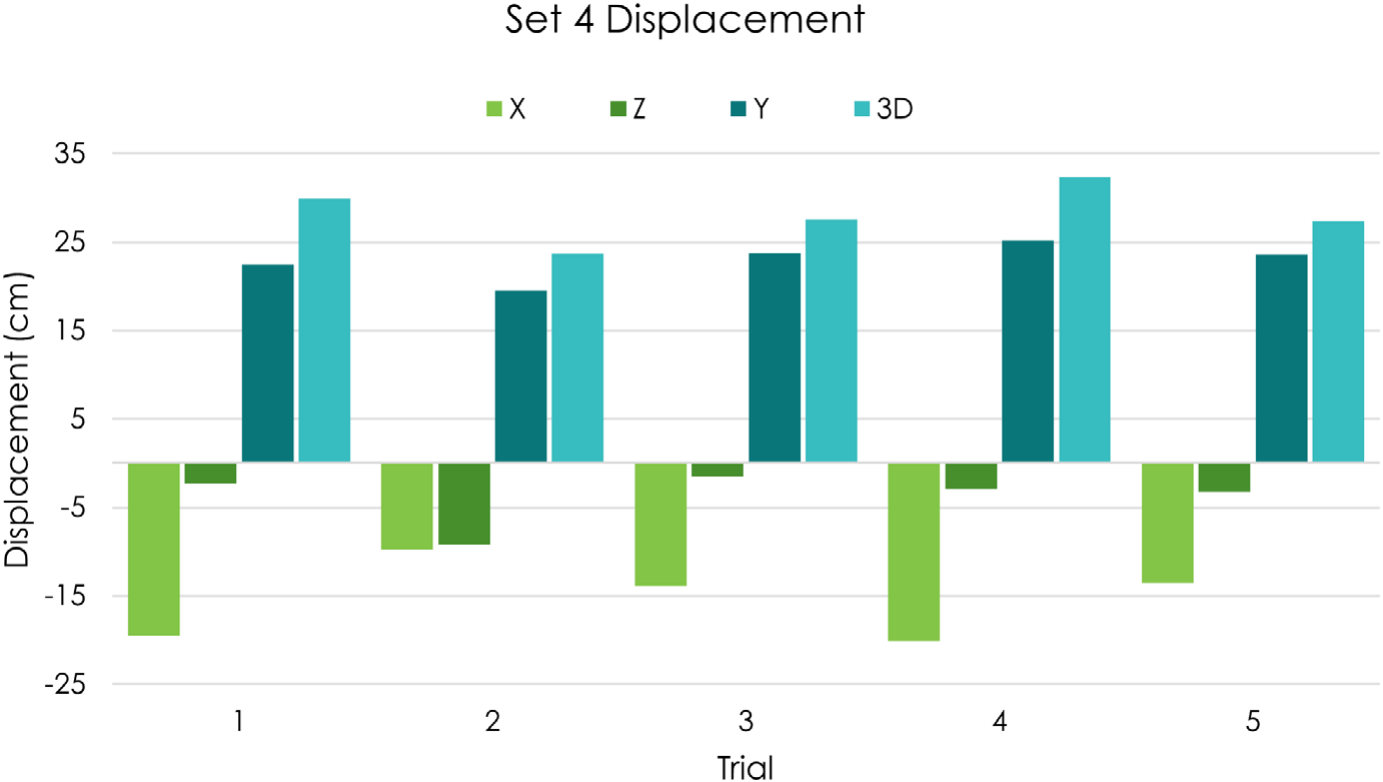
Errors between the known versus calculated values for each X, Z, Y, and 3D coordinates. Set 4 is defined as 40 cm from the wall at 45° (angled to the wall).

For each set from the rig tests, the difference between the known and calculated AO in each of the three coordinates (X, Y, and Z), along with the 3D coordinates sum, is displayed in Figures 7, 8, 9, 10. In terms of set 1 (Figure 8) and set 2 (Figure 9), the *X* errors were the lowest across each trial, and the *Y* errors were generally the highest values. In terms of set 3 (Figure 10) and set 4 (Figure 11), the *Z* errors were the lowest values across each trial, and the *Y* errors were generally the highest values.

**FIGURE 12:**
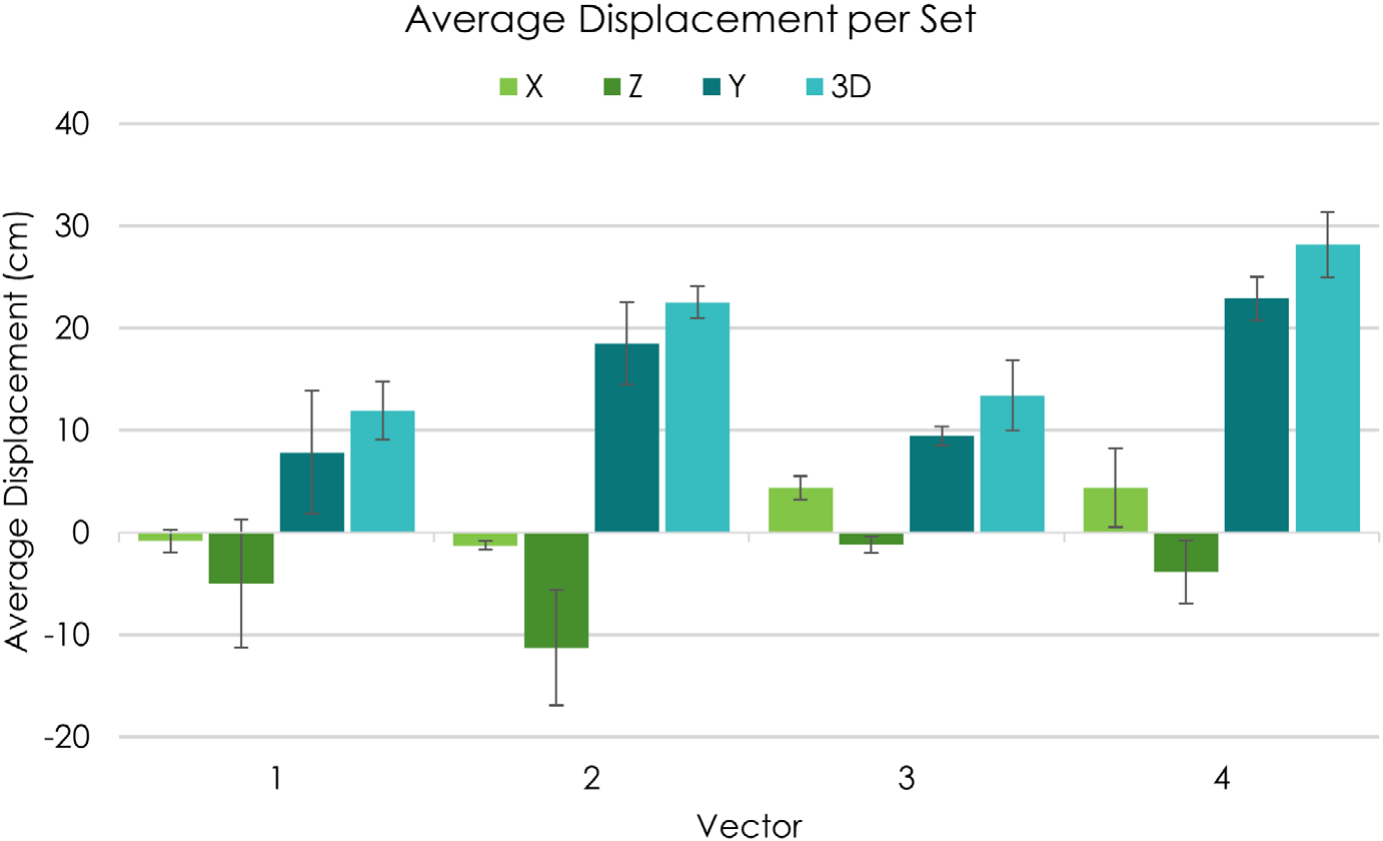
Average errors across each trial in sets 1–4 (*n* = 5), with each set being defined in Table 1. Error bars represent the standard deviation across the five trials in the set.

The average errors in each direction and the 3D coordinates sum were calculated for each of the four sets, using the values from the five trials per set (*n* = 5) (Figure 12). Set 1 and set 2 have the lowest range of values for errors in the X direction. Sets 3 and 4 have the lowest range of values for the errors in the Z direction. Notably, the errors in the Y direction generally remain the largest value across each of the four sets.

The average error values in each set, as displayed in Figure 12, were used for statistical analysis of the accuracy of the calculated AO compared to the true AO. The one‐sample *t*‐test was used to determine the statistical significance between the average mean errors and the hypothesized mean (*μ* = 0). Based on the null hypothesis, no statistical difference between the true and calculated AO indicates a hypothesized mean of zero. The results of the one‐sample *t*‐tests were outlined in Table 2.

**TABLE 2:** Results of the one‐sample *t*‐test (95% confidence interval) across each set (*n* = 5 per set).

Set	Coordinate	Average error (cm)	Min error (cm)	Max error (cm)	Standard deviation	*t*	*p*
1	X	−0.8464	−0.5180	−1.203	1.082	−1.750	0.1551
	Z	−4.980	−0.8360	−15.66	6.260	−1.779	0.1499
	Y	7.817	−2.190	12.86	6.011	2.908	0.04377
	3D	11.90	9.391	15.826	2.837	9.372	7.220E‐04
2	X	−1.285	−0.7580	−1.758	0.4064	−7.071	0.002111
	Z	−11.30	−4.972	−16.76	5.636	−4.482	0.01097
	Y	18.50	11.85	22.60	4.040	10.24	5.136E‐04
	3D	22.54	20.60	24.02	1.580	31.90	5.764E‐06
3	X	−9.074	−5.965	−16.51	4.354	−4.660	9.589E‐03
	Z	−1.177	−0.3560	−2.435	0.8055	−3.266	0.03090
	Y	9.451	7.990	10.31	0.9060	23.33	2.002E‐05
	3D	13.41	10.01	19.14	3.471	8.636	9.888E‐04
4	X	−15.36	−9.757	−20.14	4.394	−7.815	1.447E‐03
	Z	−3.853	−1.544	−9.185	3.052	−2.822	0.04771
	Y	22.90	19.52	25.13	2.106	24.31	1.698E‐05
	3D	28.16	23.68	32.34	3.222	19.55	4.040E‐05

### 3.2 Blind tests

**FIGURE 13:**
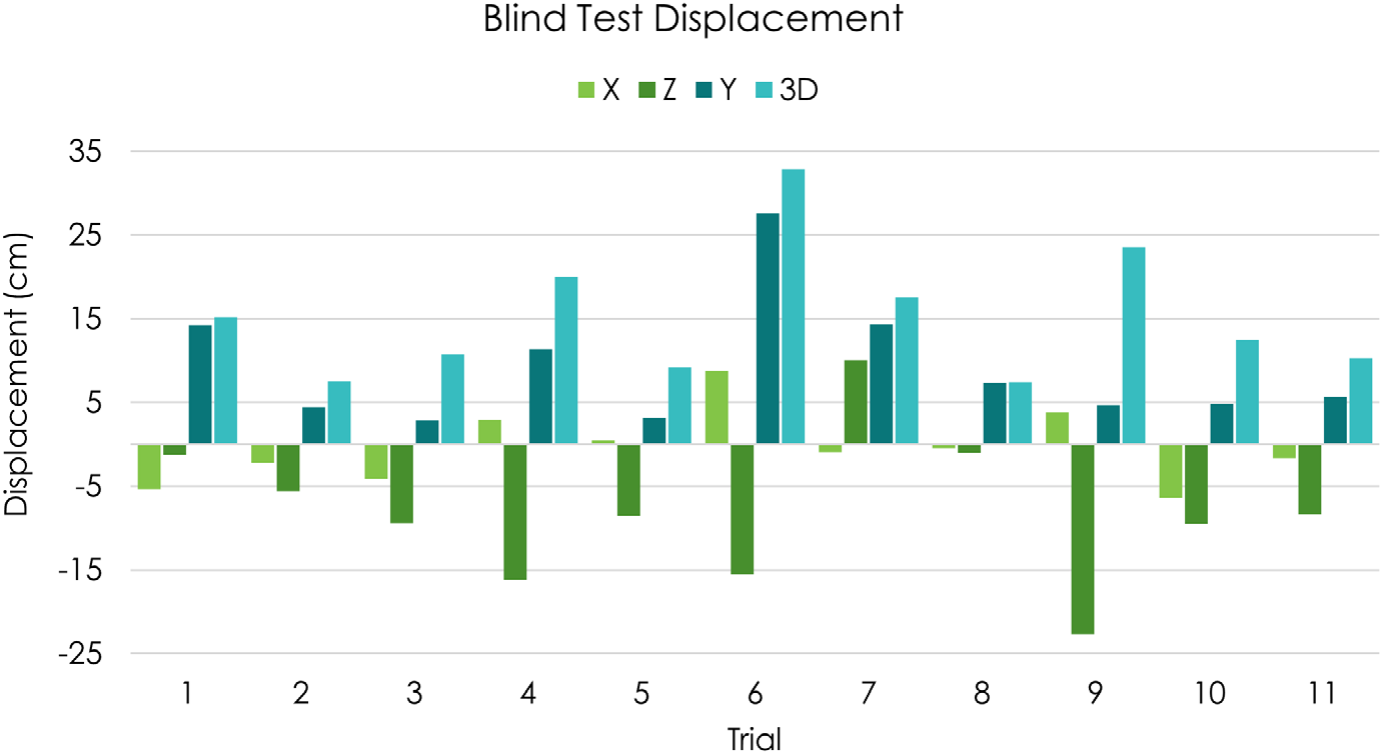
Errors between the known and calculated values for each X, Y, Z, and 3D coordinates across the 11 trials of the blind test.

**FIGURE 14:**
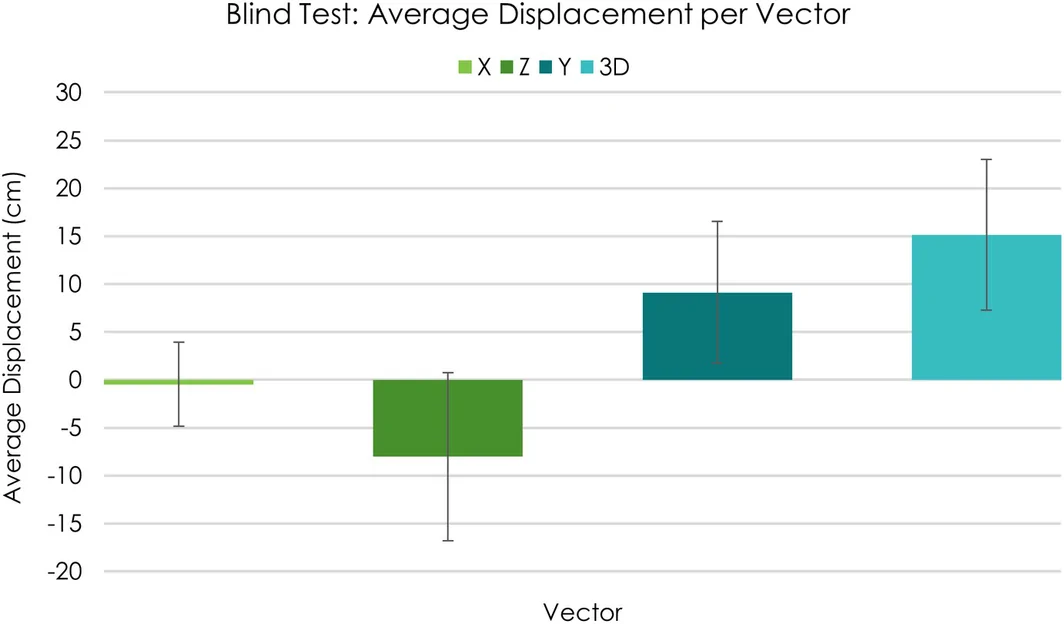
The average errors across each trial in the blind tests (*n* = 11). Error bars represent the standard deviation across the five trials in the set.

For each trial from the blind tests, the difference between the known and calculated AO in each of the three coordinates (X, Z, Y), along with the 3D coordinates sum, is displayed in Figure 13. The displayed trials vary in the volume of blood being expirated, the position of the participant relative to the wall, and the angle at which the mouth is facing from the wall. Across the trials, the errors in the X directions tends to be the lowest value, as seen in 8 of the 11 trials. The remaining 3 of the 11 trials displayed the lowest values for the errors in the Y direction. The errors in the Z direction tends the be the highest value, as seen in 7 of the 11 trials. The remaining 4 of the 11 trials displayed the highest values for the errors in the Y direction. The average calculation across each trial is plotted in Figure 14.

The errors for each coordinate, as displayed in Figure 13 and summarized in Figure 14, were used to statistically analyze the accuracy of the calculated versus true AO. The one‐sample *t*‐test was used to determine the statistical significance between the mean errors and the hypothesized mean for the X, Z, and 3D coordinates (*μ* = 0). The one‐sample Wilcoxon test was used to determine the statistical significance between the median in the Y direction compared to the hypothesized median (*x* = 0). Based on the null hypothesis, no statistical difference between the true and calculated AO indicated a hypothesized mean and median of zero. The results of the statistical tests were outlined in Table 3.

**TABLE 3:** Results of the statistical analysis (95% confidence interval) for the blind tests (*n* = 11).

Coordinate	Average errors (cm)	Min error (cm)	Max error (cm)	Standard deviation	*t*	*p*
X	−0.4797	0.4073	8.740	4.386	−0.3628	0.7243
Z	−8.028	−1.027	−22.73	8.781	−3.032	0.01263
Y	9.120	3.157	27.58	7.411	4.081	2.210E‐03
3D	15.14	7.484	32.85	7.864	6.385	5.125E‐05

Considering that the calculated values were subtracted from the true value, the coordinate system is as follows: positive values in *X* represented distances closer to the left‐hand side, positive values in Z represented distances closer to the ground, and positive values in the Y represented distances closer to the wall. Interpretations of the average errors at each coordinate across the four sets are reviewed in the discussion.

## 4 DISCUSSION

This research aimed to determine whether AO analysis can be performed on expirated patterns. For this research, HemoVision was used to determine whether the calculated AO resembled the true AO in order to identify whether the coordinates can be virtually determined for expirated patterns. Further, the difference between the true and calculated coordinates of the AO was analyzed at different distances and angles to determine whether the errors varied based on these two factors. Blind tests were conducted and analyzed to assess the quality of the AO analysis process and to determine whether similar trends in the errors existed when dealing with human participants who expirated varying volumes of air at different velocities from a range of positions and angles.

### 4.1 Rig tests

When looking at the average errors of each coordinates value across all four sets in Figure 12, set 1 and set 2 have similar trends that differ from set 3 and set 4. Interestingly, both set 1 and 2 represent the trials performed at 90° from the wall, thus positioning the mouthpiece perpendicular to the wall. Though set 1 occurs at 20 cm from the wall, and set 2 occurs at 40 cm from the wall. In the highlighted sets, the lowest error values are found in the X direction, meaning that horizontal position along the wall differs the least between the true and calculated AO (Figure 12). Set 1 presents an average *X*‐estimation error of −0.8464 cm (min = −0.5180; max = −1.203; *p* = 0.1551) and set 2 presents an average *X*‐estimation error of −1.285 cm (min = −0.7580; max = −1.758; *p* = 0.002111). This indicates that expirated patterns occurring perpendicular to the wall allow for an X‐estimation that falls within a reasonable range of the known X‐position. In this case, the X‐positions fell within an absolute value of 1.5 cm, which is a reasonable and appropriate range for investigative purposes (Table 2).

On the other hand, set 3 and set 4—positioned at 20 cm and 40 cm from the wall, respectively—represent the trials performed at 45° from the wall, thus positioning the mouthpiece angled in the X direction. In the highlighted 40 cm sets, the lowest error values were found in the Z direction, thus presenting the lowest difference between the true and calculated AO (Figure 12). Set 3 presents an average *Z*‐estimation *error* of −1.1177 cm (min = −0.3560; max = −2.435; *p* = 0.03090) and set 4 presents an average *Z*‐estimation error of −3.853 cm (min = −1.544; max = −9.185; *p* = 0.04771). This indicates that expirated patterns occurring angled to the wall in the X‐dimension allow for a Z‐estimation that falls within a reasonable range of the known Z‐position. In this case, the Z‐positions fell within an absolute value of 4 cm, which is a reasonable range for investigative purposes (Table 2).

All four sets generally have the greatest errors between the calculated Y position and the true Y position. The greatest error values are viewed in set 2 and set 4 (Figure 12; Table 2), both of which occur at 40 cm from the wall, though from 90° and 45°, respectively. When looking at sets 2 and 4 specifically, the average errors of the Y position were roughly half of the total 40 cm distance. Set 2 presents an average *Y*‐estimation error of −18.50 cm (min = 11.85; max = 22.60; *p* = 0.01097), and set 4 presents an average *Y*‐estimation error of 22.90 cm (min = 19.52; max = 25.13; *p* = 1.698E‐05). Similarly, sets 1 and 3 have average errors in the Y position that were nearly half of the total 20 cm distance. Set 1 presents an average *Y*‐estimation error of 7.917 cm (min = −2.190; max = 12.86; *p* = 0.04377) and set 3 presents an average *Y*‐estimation error of 9.451 cm (min = 7.990; max = 10.31; *p* = 2.002E‐05). This indicates a potential relationship between the calculated and true distance between the mouthpiece and surface, where the true Y‐distance is half of what the expected distance is calculated to be. While the results suggest an approximate halving of the Y position, this relationship may differ at a farther distance from the surface. Thus, further investigation would be necessary to determine the relationship between the estimated and measured Y position.

In terms of the accuracy of the calculated AO, the statistical difference must be considered. The one‐sample *t*‐test found that there was no statistical difference between the average errors and hypothesized errors in the X and Z directions for set 1 (*p* > 0.05; Table 2). With that said, a statistical difference was found between the average errors and hypothesized errors in the X and Z directions of sets 2–4, as well as the Y direction and 3D coordinates for sets 1–4 (*p* > 0.05; Table 2). Therefore, statistically accurate values in the X and Z directions are found when the mouthpiece is closest to the wall and positioned perpendicular to the surface of interest, rather than horizontally angled.

Generally, the trajectory of blood spatter is impacted by gravity and drag forces, thus resulting in a curved path. This non‐linear path may cause blood spatter to reach the greatest height of its travel before reaching the surface, thereby being positioned lower than if it had traveled a linear path [1, 3]. This results in differences in the Z direction. For simplification, this software assumes a straight‐line trajectory, which may introduce errors in *Z*‐axis estimation due to neglect of the complex fluid dynamics that contribute to a curved trajectory. While these errors are noted, they are generally minimal and considered acceptable for AO estimation.

**FIGURE 15:**
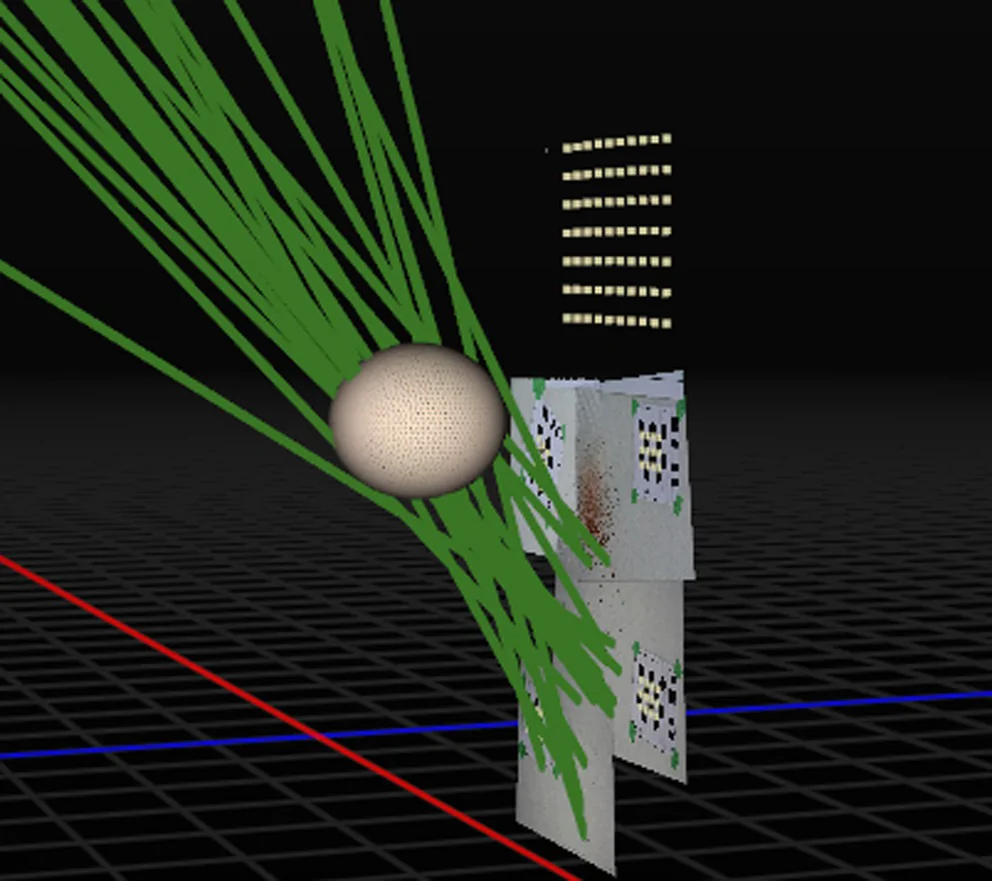
Rendered image of trial 5 from set 1, obtained through HemoVision. The path of travel of each marked blood spatter is displayed in green.

Statistically, it is complicated to determine the accuracy of AO analysis as these calculation methods simplify a complex event. For example, the current AO analysis methods assume that the blood drops travel in a straight path rather than a curved trajectory [24, 25]. This straight path can be visualized in Figure 15. By negating forces and simplifying physical events, AO analysis is susceptible to statistical errors due to discrepancies in the height coordinate. Further, the study at hand looks at expirated patterns taken from a single wall, which can further contribute to discrepancies in the X, Z, and Y coordinates. One study found that blood spatter obtained from multiple surfaces can reduce errors in the calculated AO [26]. By analyzing a single wall, the calculated AO may be prone to errors in the distance from the wall as well as the distance along the wall [4, 26]. Not only that, but the clustering radiation associated with expirated patterns may also contribute to difficulties in identifying larger stains that would otherwise provide a better estimation of the AO. This explains why comparisons between the calculated and known AO result in failed statistical tests.

Thus, investigating AO analysis still provides useful information regarding unique patterns or clustering, errors that may be associated with specific spatter patterns, and general trends within the AO analysis methods. For example, expirated spatter is associated with highly clustered and mist‐like patterns, depending on the force of air. As a result of the clustering, it may be difficult to select larger stains that can help determine the AO; however, this unique pattern can help determine the type of bloodstain pattern. The ability to characterize blood spatter at the crime scene can provide investigators with useful information. Particularly, identifying spatter as an expirated pattern can help identify the location or placement of the bleeding individual, which can aid in understanding the sequence of events. In terms of forensic significance, the AO analysis of expirated patterns produces coordinates, which can roughly place an individual within 10–20 cm of their original position [4].

Moreover, this study looked at how distance and angles may affect the AO analysis of expirated patterns. The comparisons between distances and angles can provide forensic investigators with information regarding the reliability of their AO analysis. In this study, patterns that were produced closer to the wall (at 20 cm) produced overall coordinates that were closer to the true position, in comparison to patterns produced further from the wall (at 40 cm). Among the closer sets, set 1 presented an average 3D‐estimation error of 11.90 cm (min = 9.291; max = 15.826) and set 3 presented an average 3D‐estimation error of 13.41 cm (min = 10.01; max = 19.14). Among the farther sets, set 2 presented an average 3D‐estimation error of 22.54 cm (min = 20.60; max = 24.02) and set 4 presented an average 3D‐estimation error of 28.16 cm (min = 23.68; max = 32.34). Patterns that were produced perpendicular to the wall (at 90°) produced horizontal coordinates that were closer to the true position, in comparison to patterns produced from an angle to the wall (at 45°). This refers to the average 3D‐estimations among sets 1 and 2 versus sets 3 and 4, respectively. Considering this, forensic investigators may be able to better estimate the true position of the bleeding individual based on expirated patterns produced closer or perpendicular to a surface, in this case, a single wall. Therefore, for forensic investigative purposes, BPA studies that investigate various AO analysis techniques are useful beyond statistical significance, as they provide information regarding trends, characterization, and the ability to identify a relative 3‐dimensional location within which the bleeding individual or object can be placed.

For this study, the average errors and standard deviations must be considered when determining whether the AO can appropriately be used for investigative expirated pattern analysis. The range for which the errors can fall within is relative to the total size of the location. In this case, the appropriate range was deemed as errors that fall within 10 cm of the true position [4]. When looking at the average *X*‐errors, the errors at 90° fall within a 10 cm range and thus are appropriate for investigative purposes (*μ*_set *1*_ = −0.8464 cm; *μ*_set 2_ = −1.285 cm). The *X*‐errors begin to deviate once the mouthpiece is horizontally angled to the wall, though set 3 returns promising values, which can indicate that distances closer to a surface may return more accurate *X*‐values (*μ*_set *3*_ = −9.074 cm; *μ*_set 4_ = −15.36 cm). The average *Z*‐errors across each distance and angle also fall within a 10 cm range and thus are appropriate for investigative purposes (*μ*_set *1*_ = −4.980 cm; *μ*_set 2_ = −11.30 cm; *μ*_set *3*_ = −1.177 cm; *μ*_set 4_ = −3.853 cm). The average *Y*‐errors at 20 cm from the wall fall within a 10 cm range and thus are appropriate for investigative purposes (*μ*_set *1*_ = 7.817 cm; *μ*_set 3_ = 9.451 cm). The *Y*‐errors begin to deviate at distances further from the wall, thereby cautioning investigators from using the *Y*‐value provided by HemoVision (*μ*_set *2*_ = 18.50 cm; *μ*_set 4_ = 22.90 cm). Therefore, AO analysis for expirated patterns generally returns values falling within a reasonable 10 cm range in the X and Z directions, meaning that investigators could use BPA techniques to appropriately identify the AOC as opposed to the AO.

### 4.2 Blind test

The blind tests vary in factors such as volume of air, velocity of air, volume of blood, and presence of fluids being mixed with the blood (i.e., saliva, phlegm), which may impact the path of travel that the blood takes through the air. Further, the participants expirated blood onto the surface from various angles and positions—notably, at various heights and distances from the wall and with different forces. As a result, the expirated patterns created through the blind tests are diverse and display characteristics that may differ from the patterns created by the mechanical rig. For example, the presence of saliva and/or phlegm creates string‐like patterns as opposed to a mist‐like pattern formed by high velocity dispersion of blood. Further, minimal movement during a human coughing motion may present minor effects in velocities or dispersion. Therefore, the blind test allows for the assessment of human participants and the realistic depictions of expirated patterns caused by voluntary coughs from adult individuals.

**FIGURE 16:**
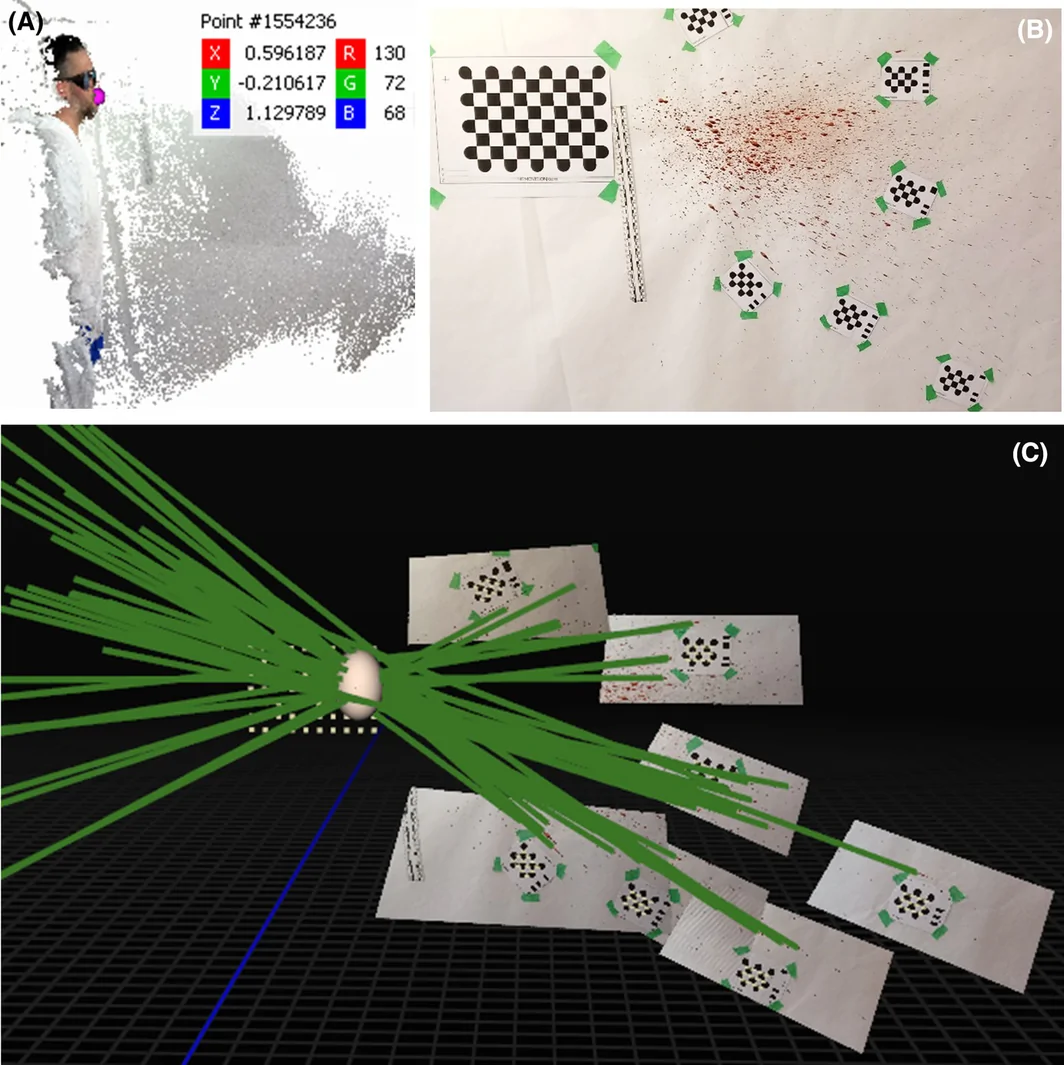
Trial 8 of the blind tests. (A) 3D scan of the participant, taken perpendicular to the wall. (B) Overall view of the resultant pattern. (C) Rendering of AO estimation through HemoVision, with AO depicted in white.

In terms of specific blind test trials, trials 8 and 2 resulted in two of the most accurate estimations of the AO (Figure 13). Trial 8 had the lowest 3D errors and housed one of the lowest errors for the X and Z coordinates (*μ*_X_ = −0.4363 cm; *μ*_Z_ = −1.027 cm; *μ*_Y_ = 7.293 cm; *μ*_3D_ = 7.378 cm). Based on the AO analysis through HemoVision, trial 8 had a greater array of elliptical stains from upward and downward angles, which allows for more stains being marked and a better estimation of the AO. Similarly, trial 2 had a spread of elliptical patterns from the top and bottom halves of the surface, which would allow for elliptical stains being marked from an upward and downward angle and thus, result in a better estimation for the AO (*μ*_X_ = −2.234 cm; *μ*_Z_ = −5.597 cm; *μ*_Y_ = 4.439 cm; *μ*_3D_ = 7.484 cm). When looking at the 3D scans for trials 8 and 2, both trials were done with the participant facing an acute angle from the wall in the horizontal direction. That is, the orifice was nearly parallel to the wall at a close distance to the surface, as shown in Figure 16, which displays a 3D scan of trial 8. The resulting patterns, observed through HemoVision, displayed an arrangement of elliptical stains from upward and downward angles—thus allowing for a better estimation of the Z and Y coordinates. The resulting AO estimations were found to be the most accurate in comparison to the other blind test trials.

**FIGURE 17:**
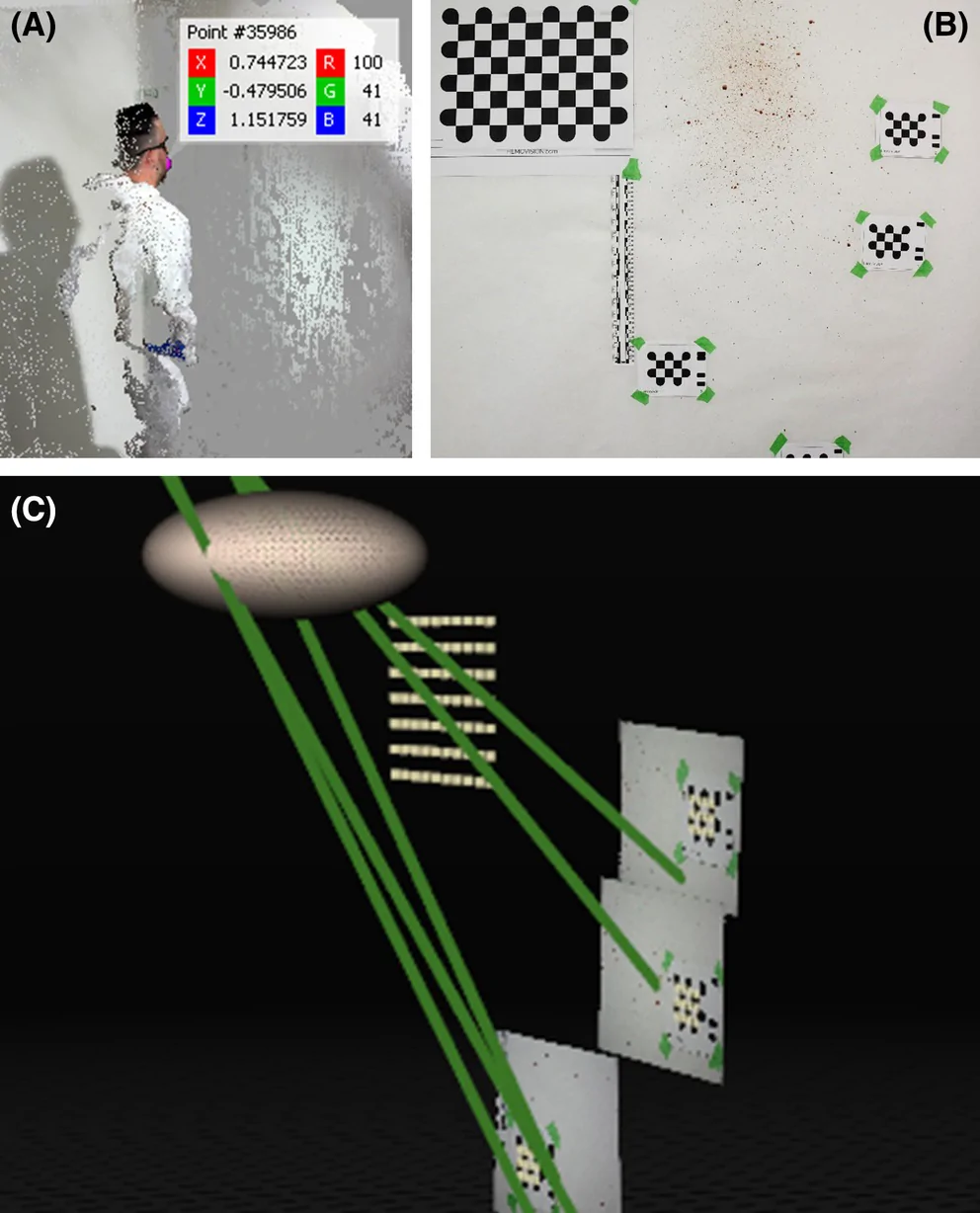
Trial 6 of the blind tests. (A) 3D scan of the participant, taken angled from the wall. (B) Overall view of the resultant pattern. (C) Rendering of AO estimation through HemoVision, with AO depicted in white.

When looking at the other blind tests, the estimations of the Y and Z coordinates generally display the greatest discrepancies. Trial 6 displays the greatest errors in the Y coordinates and the 3D coordinates sum (*μ*_Y_ = 27.58 cm; *μ*_3D_ = 32.85 cm; Figure 13). When analyzing the pattern formed by trial 6, there were many circular blood drops as opposed to elliptical stains, which hindered the AO analysis. Interestingly, based on the 3D scans, the participant was faced perpendicular to the wall when expirating the blood for trial 6, which would contribute to a greater number of circular stains (Figure 17). Elliptical stains are vital for the AO analysis process, as they indicate information regarding the angle and path of travel; thus, a lack of elliptical stains results in a greater variance in the AO estimation. Trial 9 houses the second greatest 3D coordinates sum of the errors and further has the greatest errors in the Z direction (*μ*_Z_ = −22.73 cm; *μ*_3D_ = 23.52 cm; Figure 13). When analyzing the patterns formed by trial 9 through HemoVision, many of the elliptical stains were found near the bottom of the paper, with very few stains coming from an upward angle. Interestingly, based on the 3D scans, the participant for trial 9 was facing a downward angle when expirating the blood onto the surface. As a result, many notable elliptical stains were found towards the bottom‐half of the surface, which resulted in a greater estimation of the Z coordinates.

To get a better understanding of the estimations of each of the coordinates, the average errors were analyzed across the 11 blind tests in Figure 14. As stated, and further seen in Figure 14, the errors in the X position were the lowest value, which indicates that the estimation of the X coordinates is the closest to the true value (*μ*_X_ = −0.4798 cm; min_X_ = −0.4363 cm; max_X_ = 8.740 cm). Statistically, the one‐sample *t*‐test found no significant differences between the known and estimated average position for the X position (*p* > 0.05; Table 3). In comparison, the estimations for the Z and Y positions display the greatest difference from the true position, with errors of −8.028 cm (min_Z_ = −1.027 cm; max_Z_ = 22.73 cm) and 9.120 cm (min_Y_ = 3.157 cm; max_Y_ = 27.58 cm), respectively (Table 3). This signifies that the estimations for Z and Y may pose issues in terms of accurate AO analysis, as this estimation results in *Z* values that are at a greater height than the true position and *Y* values that are perpendicularly closer to the surface than the true position. Statistically, the Z and Y positions displayed a statistically significant difference between the known and estimated mean (*p* < 0.05; Table 3). Therefore, the AO analysis process for the blind tests displayed a more accurate estimation of the X position as opposed to the Z or Y position.

The blind test is to evaluate the quality of the AO analysis by analyzing the expirated patterns produced by human participants who were positioned at various angles and distances. Further, human participants allowed for better assessment of realistic expirated patterns as these patterns were influenced by contributing factors such as the presence of other fluids, differing volumes and velocities of air, and differing cough types. Ultimately, it was found that the estimation of the X position was the closest to the true *X* value across each 8 of the 11 trials. The estimations of the Z and Y positions generally displayed the greatest difference from the true values, which contributed to a larger 3D coordinates sum of the errors. Based on the AO analysis process itself, more accurate estimations of the 3D position were found in stains with a diverse array of elliptical stains found in the upper and lower halves of the surface. That is, when elliptical stains are found at upwards and downwards angles, the assessment of the Z and Y positions tended to be more accurate.

## 5 CONCLUSION

The results from this study show that statistically, the AO may not be accurately identified for expirated patterns. Further, the angle and distance at which the mouth is facing from the surface can also influence the AO analysis process when dealing with expirated patterns. In terms of the angle, patterns produced perpendicular to the wall return more accurate horizontal values (*X*) than horizontally angled positions. In terms of the distance, patterns produced closer to the wall return more accurate vertical values (*Z*) along with more accurate distance values (*Y*). The notable difference in the Y direction cautions investigators from appropriately estimating the distance between the orifice and surface through AO analysis processes. Therefore, the AO of expirated patterns may not be accurately identified. Although the AO may not be appropriately determined, the *X* and *Z* values may be used to identify the AOC of expirated patterns and may still be useful to tell the difference between whether the victim was standing, kneeling, or lying down.

A limitation of expirated pattern analysis is the diversity in characteristics posed by patterns created by different orifices [12]. This study focused on the mouth as the orifice of origin, using a synthetic mouthpiece created through 3D‐printing. While the design of the mouthpiece was based on literature to mimic aspects of human oral anatomy, it does not fully replicate complex dynamics involved with human expirations [13, 15, 17, 18, 20, 21]. Although human participants were involved to further explore human‐based patterns, it is recommended to use anatomically accurate mouthpieces to improve realism. Recent studies have made advancements to 3D‐print oral models that account for the anatomy of the tongue, oral cavity, closed soft palate, and pharynx [27]. As such, future research should explore the use of anatomically accurate mouthpieces, accounting for both oral and nasal cavities, to improve the accuracy of the resulting patterns [27]. Considering the complexities of human anatomy, it is further recommended that researchers conduct human‐based experiments to assess differences in resulting expirated patterns.

Similarly, another limitation of expirated pattern analysis is contributed by the force at which air passes in order to create such patterns [12]. This study focused on coughing of blood produced by an adult in order to define a set of parameters that may limit confounding variables. Thus, these results may not be suitable for other mechanisms of expirated patterns. Future research would benefit from looking into the difficulty and accuracy of analyzing different types of expirated patterns through virtual software, thus testing varying orifices and forces of air. As seen in the blind tests, it would benefit future research to work with human participants who can contribute to confounding variables, such as fluids being mixed with blood and various types of coughs at differing degrees of force.

Moreover, the parameters defining the mechanism of expirated pattern, volume and velocity of air, differ among adults. Another limitation would be the volume and velocity used for this study. Future research would benefit from testing how the volume and velocity of air in a cough influence the types of stains produced through expirated patterns. Further, studies should assess whether AO analysis methods, both traditional and virtual, are suitable for such stains.

## FUNDING INFORMATION

University of Toronto Mississauga Experiential Learning Bursary. Presented at the International Association of Bloodstain Pattern Analysis Conference, October 9, 2024, in Kansas City, MO.

## CONFLICT OF INTEREST STATEMENT

The authors disclose that the co‐author is the developer of the application of Recon‐3D.

## Data Availability

The data that support the findings of this study are available from the corresponding author upon reasonable request.

## References

[1] James SH , Kish PE , Sutton TP . Principles of bloodstain pattern analysis: theory and practice. Boca Raton, FL: CRC Press/Taylor & Francis; 2005.

[2] AAFS Standards Board . Terms and definitions in bloodstain pattern analysis. Colorado Springs, CO: AAFS Standards Board, LLC; 2017 Report No.: ASB Technical Report 033.

[3] Reynolds J , Liscio E . Determining the accuracy of area of origin for impact patterns found on horizontal surfaces. J Assoc Crime Scene Reconstr. 2022;26:1–14.

[4] Hakim N , Liscio E . Calculating point of origin of blood spatter using laser scanning technology. J Forensic Sci. 2015;60(2):409–417. 10.1111/1556-4029.12639 25676744

[5] Illes M , Boués M . Investigation of a model for stain selection in bloodstain pattern analysis. J Can Soc Forensic Sci. 2011;44(1):1–12. 10.1080/00085030.2011.10768137

[6] Rosina J , Kvasnak E , Suta D , Kolárová H , Malek J , Krajci L . Temperature dependence of blood surface tension. Physiol Res Acad Sci Bohemoslov. 2007;56(Suppl 1):S93–S98. 10.33549/physiolres.931306 17552890

[7] Griffiths G , Liscio E , Guryn H , Le Q , Northfield D , Williams GA . Inter‐observer error for area of origin analysis using FARO zone 3D. Sci Justice. 2021;61(3):291–298. 10.1016/j.scijus.2021.02.006 33985677

[8] de Bruin KG , Stoel RD , Limborgh JCM . Improving the point of origin determination in bloodstain pattern analysis. J Forensic Sci. 2011;56(6):1476–1482. 10.1111/j.1556-4029.2011.01841.x 21790597

[9] Joris P , Jenar E , Moermans R , Van de Voorde W , Vandermeulen D , Claes P . Bloodstain impact pattern area of origin estimation using least‐squares angles: a HemoVision validation study. Forensic Sci Int. 2022;333:111211. 10.1016/j.forsciint.2022.111211 35172260

[10] Vitiello A , Di Nunzio C , Garofano L , Saliva M , Ricci P , Acampora G . Bloodstain pattern analysis as optimisation problem. Forensic Sci Int. 2016;266:e79–e85. 10.1016/j.forsciint.2016.06.022 27462014

[11] Joris P , Develter W , Jenar E , Suetens P , Vandermeulen D , Van de Voorde W , et al. HemoVision: an automated and virtual approach to bloodstain pattern analysis. Forensic Sci Int. 2015;251:116–123. 10.1016/j.forsciint.2015.03.018 25911495

[12] Donaldson AE , Walker NK , Lamont IL , Cordiner SJ , Taylor MC . Characterising the dynamics of expirated bloodstain pattern formation using high‐speed digital video imaging. Int J Legal Med. 2011;125(6):757–762. 10.1007/s00414-010-0498-5 20668870

[13] Zanella A , Cressoni M , Ferlicca D , Chiurazzi C , Epp M , Rovati C , et al. Spatial orientation and mechanical properties of the human trachea: a computed tomography study. Respir Care. 2015;60(4):561–566. 10.4187/respcare.03479 25492954

[14] Safshekan F , Tafazzoli‐Shadpour M , Abdouss M , Shadmehr MB . Mechanical characterization and constitutive modeling of human trachea: age and gender dependency. Materials. 2016;9(6):456. 10.3390/ma9060456 28773579 PMC5456771

[15] Mahajan S , Mahajan A , Lalit M , Verma P . Morphometry of adult human trachea and its clinical implications: a cadaveric study in northern India. J Clin Diagn Res. 2022;16(7):AC01–AC05. 10.7860/JCDR/2022/55145.16574

[16] Otoch JP , Minamoto H , Perini M , Carneiro FO , de Almeida Artifon EL . Is there a correlation between right bronchus length and diameter with age? J Thorac Dis. 2013;5(3):306–309. 10.3978/j.issn.2072-1439.2013.03.12 23825764 PMC3698291

[17] Brandimore AE , Troche MS , Huber JE , Hegland KW . Respiratory kinematic and airflow differences between reflex and voluntary cough in healthy young adults. Front Physiol. 2015;6:284. 10.3389/fphys.2015.00284 26500560 PMC4598583

[18] Hegland KW , Troche MS , Davenport PW . Cough expired volume and airflow rates during sequential induced cough. Front Physiol. 2013;4:16I7. 10.3389/fphys.2013.00167 23847546 PMC3701804

[19] Wanger J , Clausen JL , Coates A , Pedersen OF , Brusasco V , Burgos F , et al. Standardisation of the measurement of lung volumes. Eur Respir J. 2005;26(3):511–522. 10.1183/09031936.05.00035005 16135736

[20] Feinstein AJ , Zhang Z , Chhetri DK , Long J . Measurement of cough aerodynamics in healthy adults. Ann Otol Rhinol Laryngol. 2017;126(5):396–400. 10.1177/0003489417694912 28397558 PMC10241208

[21] Chang AB . The physiology of cough. Paediatr Respir Rev. 2006;7(1):2–8. 10.1016/j.prrv.2005.11.009 16473809

[22] Carter AL . The directional analysis of bloodstain patterns theory and experimental validation. J Can Soc Forensic Sci. 2001;34(4):173–189. 10.1080/00085030.2001.10757527

[23] Geoghegan PH , Laffra AM , Hoogendorp NK , Taylor MC , Jermy MC . Experimental measurement of breath exit velocity and expirated bloodstain patterns produced under different exhalation mechanisms. Int J Legal Med. 2017;131(5):1193–1201. 10.1007/s00414-017-1545-2 28154922

[24] Attinger D , Comiskey PM , Yarin AL , Brabanter KD . Determining the region of origin of blood spatter patterns considering fluid dynamics and statistical uncertainties. Forensic Sci Int. 2019;298:323–331. 10.1016/j.forsciint.2019.02.003 30974388

[25] Attinger D , Moore C , Donaldson A , Jafari A , Stone HA . Fluid dynamics topics in bloodstain pattern analysis: comparative review and research opportunities. Forensic Sci Int. 2013;231(1):375–396. 10.1016/j.forsciint.2013.04.018 23830178

[26] Lee R , Liscio E . The accuracy of laser scanning technology on the determination of bloodstain origin. J Can Soc Forensic Sci. 2016;49(1):38–51. 10.1080/00085030.2015.1110918

[27] Lee K‐M , Rhee K‐J , Seo Y , Lee S‐Y . Development of a quantitatively controlled expirated bloodstain generation system using an anatomically‐informed oral model. Forensic Sci Int. 2025;377:112653. 10.1016/j.forsciint.2025.112653 40946383

